# Prevalence and Characteristics of the Course of Dysphagia in Hospitalized Older Adults

**DOI:** 10.3390/nu15204371

**Published:** 2023-10-15

**Authors:** Ayano Nagano, Masami Onaka, Keisuke Maeda, Junko Ueshima, Akio Shimizu, Yuria Ishida, Shinsuke Nagami, Shuzo Miyahara, Keiji Nishihara, Akiyuki Yasuda, Shosuke Satake, Naoharu Mori

**Affiliations:** 1 Department of Nursing, Nishinomiya Kyoritsu Neurosurgical Hospital, 11-1, Imazuyamanaka-cho, Nishinomiya 663-8211, Japan; aya.k.nagano@gmail.com; 2 Department of Palliative and Supportive Medicine, Graduate School of Medicine, Aichi Medical University, 1-1, Yazakokarimata, Nagakute 480-1195, Japan; j.ueshima@gmail.com (J.U.); a.shimizu.diet@gmail.com (A.S.); nmori@aichi-med-u.ac.jp (N.M.); 3 Department of Geriatric Medicine, National Center for Geriatrics and Gerontology, Obu 474-8511, Japan; onaka16@ncgg.go.jp (M.O.); s-miyahara@ncgg.go.jp (S.M.); nishi125@ncgg.go.jp (K.N.); a-yasuda@ncgg.go.jp (A.Y.); satakes@ncgg.go.jp (S.S.); 4 Nutrition Therapy Support Center, Aichi Medical University Hospital, 1-1, Yazakokarimata, Nagakute 480-1195, Japan; 5 Department of Nutritional Service, NTT Medical Center Tokyo, 5-9-22, Higashi-Gotanda, Shinagawa-ku, Tokyo 141-8625, Japan; 6 Department of Food and Health Science, Faculty of Health and Human Development, The University of Nagano, Nagano 380-8525, Japan; 7 Department of Nutrition, Aichi Medical University Hospital, 1-1, Yazakokarimata, Nagakute 480-1195, Japan; 8 Department of Speech Language Pathology and Audiology, Faculty of Rehabilitation, Kawasaki University of Medical Welfare, Kurashiki 701-0193, Japan; shinsuke.nagami.0514@gmail.com

**Keywords:** swallowing disorder, sarcopenia, nutrition, rehabilitation, oral care

## Abstract

Sarcopenic dysphagia (SD) is an emerging concern in geriatric medicine. This study aimed to identify the prevalence, progression, and distinct attributes of SD in patients in the Department of Geriatric Medicine. Older adult patients admitted between 2021 and 2022 were enrolled. The department conducts a comprehensive geriatric assessment (CGA) combined with a multidisciplinary team-based intervention, setting the standard for medical care. We diligently assessed the occurrence and development of dysphagia at both the admission and discharge phases. Of the 180 patients analyzed (38.9% male, mean age 86.0 ± 6.6 years), 22.8% were diagnosed with SD at admission, thrice the rate of other dysphagia variants. Only one patient had new-onset dysphagia during hospitalization, attributed to SD. Patients with SD showed a better recovery rate (18.9%) than those with other dysphagia types. Patients with diminished swallowing capacity had compromised nutritional profiles, diminished energy and protein consumption, and extended fasting durations. Although sarcopenia is a prevalent inducer of dysphagia in older adults, an integrated approach in geriatric medicine involving rehabilitation, nutrition, and dentistry is pivotal. Strategies rooted in CGA promise potential for addressing dysphagia.

## 1. Introduction

Dysphagia is a common problem in older adults [1], predominantly because of comorbid health conditions [2]. A recent study of geriatric patients (age ≥ 65 years) admitted to a Danish acute medical unit revealed a 43.1% prevalence of dysphagia [3], while another study found it to be 30.7% in hospitalized patients [4]. Geriatric patients are at a higher risk of aspiration due to cognitive impairment, neuromuscular dysfunction, and dysphagia [5,6]. In stroke patients, the risk of developing pneumonia is higher in those with dysphagia, and the risk of pneumonia is even higher in patients who are observed to aspirate [7]. These problems lead to the prolongation of hospitalization, an increased burden on public healthcare [7,8], and even adversely impact prognosis and rehabilitation strategies [9]. Dysphagia is common in frail older people [10], and due to its potential to become a significant health issue, careful attention and care are necessary. Unfortunately, not all medical practitioners fully recognize the importance of dysphagia and its risks. In busy environments or when prioritizing other diseases, the screening and assessment of dysphagia may not be conducted appropriately [4].

In recent years, several studies have highlighted a potential relationship between sarcopenia and dysphagia [11,12]. Sarcopenia is a common health challenge in older people and can be considered part of geriatric syndromes [13]. Muscle loss and muscle weakness are associated with limitations in the daily lives of older people and can contribute to multiple geriatric syndromes. Muscle loss due to sarcopenia may also coincide with muscle loss associated with swallowing. Swallowing involves the muscles of the throat and mouth, and weakness of these muscles may affect the ability to properly swallow food and liquids. Therefore, as sarcopenia progresses, the likelihood of problems with swallowing increases. These problems have recently been highlighted as feeding and swallowing problems in sarcopenia.

Sarcopenic dysphagia (SD) is characterized by sarcopenia-induced swallowing disorder and a loss of swallowing muscle mass and function [14,15]. Its diagnosis excludes obvious etiologies, such as stroke. Maeda et al. reported that 76.8% and 30% of hospitalized older patients had sarcopenia and dysphagia, respectively [16]. This study further revealed that in patients with sarcopenia, the ability to perform activities of daily living (ADL, assessed using the Barthel Index) and skeletal muscle mass index (SMI) were independent risk factors for dysphagia. Wakabayashi et al. reported that sarcopenia could cause dysphagia in 32% of inpatients who underwent dysphagia rehabilitation [17]. They also suggested that SD had a worse prognosis than dysphagia due to other diseases [17]. We have previously reported that 13.5% of older female patients who underwent proximal femoral fracture surgery developed dysphagia associated with advanced sarcopenia [18]. Since SD is a relatively recent concept that requires more research and standardized diagnostic criteria, the prevalence data for this specific condition are not widely available.

SD emerges as a critical concern in the context of aging populations, yet comprehensive data on its prevalence and progression remain limited. Understanding the interplay between sarcopenia and dysphagia is paramount, given their individual implications for the health and well-being of older adults. Both conditions can significantly compromise quality of life, independence in daily activities, and overall health outcomes. Consequently, addressing these challenges comprehensively through early diagnosis and effective management is imperative.

This study aims to fill the existing knowledge gap by investigating the prevalence and trajectory of SD in geriatric patients admitted to a specialized medical center for the elderly. By examining the course and characteristics of SD during hospitalization, we seek to shed light on the evolution of this condition in a vulnerable population. Our findings hold great potential in several domains. Firstly, they can enable the identification of individuals at high risk for SD at an early stage, allowing for timely preventive interventions. This proactive approach can significantly improve patient outcomes and reduce healthcare burdens associated with the management of advanced SD. Secondly, insights into the course and specific characteristics of SD may pave the way for the development of more effective therapeutic strategies. Tailoring interventions to the unique needs and challenges posed by SD can enhance treatment efficacy, ultimately benefiting the overall health and well-being of older adults. In summary, this study seeks to provide a comprehensive understanding of the prevalence and course of SD in geriatric patients.

## 2. Materials and Methods

### 2.1. Study Design and Participants

This cohort study focused on geriatric inpatients admitted to the Department of Geriatrics Medicine at the National Center for Geriatrics and Gerontology in Aichi, Japan, spanning from 1 January 2021, to 31 December 2022. The Department of Geriatrics Medicine employs a comprehensive geriatric assessment (CGA) approach characterized by multidisciplinary evaluation and team-based medical intervention [19]. CGA is a systematic evaluation that encompasses the medical conditions of older patients and evaluates their activities of daily living (ADL), mental and psychological functions, social and economic functions, and overall quality of life (QOL) [20]. The general steps of a CGA include initial assessment, diagnosis, and intervention planning. In the initial assessment, information is collected about the patient’s background, medical history, and home environment. This includes a multifaceted evaluation involving physical assessment, cognitive assessment, psychosocial assessment, nutritional assessment, medication review, and functional assessment. Based on these assessments, health issues and risks are identified, leading to the formulation of a diagnosis and intervention plan. Regular follow-ups and reassessment are conducted to monitor the effectiveness of interventions and make necessary adjustments in response to changes in the patient’s condition. The care provided in geriatric medicine emphasizes close collaboration between rehabilitation, nutrition, and dentistry, as outlined in Table 1.

To ensure this study’s focus on relevant participants, certain exclusion criteria were applied. Patients with planned hospital stays of less than two days, those who did not provide consent for inclusion in the study, individuals with a life expectancy of less than one month, patients readmitted within three months, and those with incomplete or missing data were excluded from the analysis.

Ethical considerations were paramount throughout this study. Approval for the research was obtained from the Institutional Review Board of the National Center of Geriatrics and Gerontology (Approval ID: 1388, Approval Date: 5 August 2022). The study strictly adhered to ethical guidelines, including obtaining informed consent from both the patients and their legal guardians, in accordance with the principles outlined in the Declaration of Helsinki [21].

### 2.2. Baseline Parameters

Patient information collected during hospitalization included age, sex, disease presentation, comorbidities, level of nursing care required, food texture before admission and at discharge, nutritional risk at admission, number of days without oral intake, energy and protein intake in the first 5 days after admission (5-day average), and length of hospital stay. The level of nursing care required was assessed according to the Degree of Independence of Disabled Elderly Persons in Performing Activities of Daily Living criteria proposed by the Ministry of Health and Welfare, Japan [22]. This degree of independence is classified as Rank 0 = no disability and completely independent in ADL; Rank 1 = some disability, but mostly independent in ADL and can go outside unassisted; Rank 2 = mostly independent in home-based ADL but requires assistance to go outside; Rank 3 = requiring considerable assistance in home-based ADL and uses a wheelchair; Rank 4 = bedridden, requiring assistance for most ADL. The Charlson Comorbidity Index (CCI) and Mini Nutritional Assessment-Short Form (MNA-SF) were used to assess comorbidity and nutritional risk, respectively. CCI is a widely used medical tool for assessing the burden of comorbidities (the presence of two or more chronic medical conditions) in patients. The CCI assigns a score to specific chronic medical conditions based on their severity and potential impact on mortality [23]. Each condition is assigned a weight or score, which is then summed up to calculate an overall comorbidity score for a patient. The higher the score, the greater the burden of comorbidities and the higher the predicted mortality risk. The MNA-SF is a nutritional risk screening tool for older adults and ranges from 0 to 14 points, with a score of 11 or less indicating a nutritional risk [24]. Energy and protein intake were assessed by the Nutrition Support Team, a multidisciplinary team of trained nutrition professionals. The Barthel Index is a common tool for assessing ADL, particularly in older adults and those with physical disabilities. This index is widely employed in the medical field and rehabilitation processes to measure a patient’s level of independence in their day-to-day activities. Scores typically range from 0 points, indicating complete dependence, to 100 points, signifying complete independence.

### 2.3. Sarcopenia Diagnosis Methods

SMI was calculated from the appendicular skeletal muscle mass based on dual X-ray absorptiometry (Lunar iDXA; GE Healthcare, Chicago, IL, USA) divided by the height [m] squared. Handgrip strength was measured in the sitting position with a 90-degree elbow bend using a Jamar-type instrument (MG-4800, MORITOH Co., Aichi, Japan). The highest right and left-hand values were recorded in kilograms to one decimal place. The chair stand test was conducted to assess physical function. The chair stand test is a simple assessment used to measure lower body strength and functional mobility in individuals, particularly in older adults [25]. It can help identify declines in lower body strength and mobility, monitor progress during physical therapy or exercise programs, and determine a person’s risk of falls or functional limitations. Sarcopenia was diagnosed according to the latest criteria from the Asian Working Group for Sarcopenia [13], which required a low SMI (<7.0 kg/m^2^ for men and <5.4 kg/m^2^ for women), a decreased handgrip strength (<28 kg for men and <18 kg for women) and/or a decreased chair stand test time ≥ 12 s.

### 2.4. Dysphagia and Sarcopenic Dysphagia Diagnosis Methods

Swallowing function was assessed using the Functional Oral Intake Scale (FOIS), a validated measure. FOIS [26] was scored from 1 (poor functioning) to 7 (normal) based on the patient’s ability to consume food and liquids orally. Based on the FOIS score, patients were classified into Level 1, could not consume anything by mouth; Level 2, tube dependent with minimal attempts of food or liquid; Level 3, tube dependent with consistent oral intake of food or liquid; Level 4, consumed an oral diet of single consistency; Level 5, consumed an oral diet of multiple consistencies requiring special preparation or compensation; Level 6, consumed an oral diet of multiple consistencies without special preparation but with specific food limitations; and Level 7, consumed an oral diet with no restrictions. FOIS ≤ 5 was defined as dysphagia. The presence or absence of dysphagia and the determination of FOIS were made by a multidisciplinary team consisting of physicians with expertise in dysphagia and speech-language pathologists. In addition to screening tests and evaluations during actual meal situations, detailed swallowing function evaluations were conducted as needed using the videofluoroscopic swallowing study (VFS) and functional endoscopic evaluation of swallowing (FEES).

SD was determined based on the algorithm described by Mori et al. [27]. We targeted individuals aged ≥65 who were able to follow instructions, assessing for the presence of systemic sarcopenia. If systemic sarcopenia was detected, we then evaluated for a decline in swallowing functions. In cases of dysfunction, we assessed for evident causes of dysphagia (such as stroke, traumatic brain injury, neuromuscular diseases, and head and neck cancer). Those with obvious causes were excluded. However, even if there was a cause for the dysphagia, if sarcopenia was considered the primary factor, they were still included as participants. When sarcopenia is thought to be the primary cause of the dysphagia, the decline in muscle strength of the swallowing-related muscle groups was assessed using tongue pressure. If the tongue pressure was above 20 kPa and there was no decline in swallowing-related muscles, or if tongue pressure could not be measured, it was categorized as “possible sarcopenic dysphagia”. If the tongue pressure was 20 kPa or below and there was a decline in the strength of swallowing-related muscles, it was considered “provable sarcopenic dysphagia”. In this study, regarding the cause of dysphagia, multifaceted diagnostic reasoning was conducted for each patient based on a prospective CGA. In this context, the decision on whether the dysphagia was caused by a stroke was based on the opinions of geriatricians and the medical staff at the National Center. Specifically, we meticulously examined the patient’s dietary situation before hospitalization, the onset of feeding and swallowing disorders, and the surrounding environment at that time, from a medical perspective. SD was diagnosed based on dysphagia at admission and discharge, using information on sarcopenia diagnosis at admission. Diseases causing dysphagia were determined based on patient history and the primary disease on admission.

### 2.5. Statistical Analysis

The prevalence of SD and other types of dysphagia was also determined. Patients were classified into two groups based on the presence or absence of dysphagia at admission and during hospitalization. Descriptive statistics were performed to calculate *p*-values and effect sizes. Differences in recovery rates between SD and other types of dysphagia were analyzed using McNemar’s test. Continuous and ordinal data are presented as mean ± standard deviation (SD) and median [25, 75 percentiles], respectively, and the differences were analyzed using the t-test and Mann–Whitney U-test. Categorical data are expressed as incidents and percentages, and differences were analyzed using Fisher’s exact test. *p*-values ≤ 0.05 were considered significantly different, and effect sizes of <0.2, 0.2–0.4, 0.4–0.6, and ≥0.6 were considered no effect, low, moderate, and high, respectively. The statistical tests were performed using SPSS 26.0 software (IBM Japan, Tokyo, Japan).

## 3. Results

**Figure 1:**
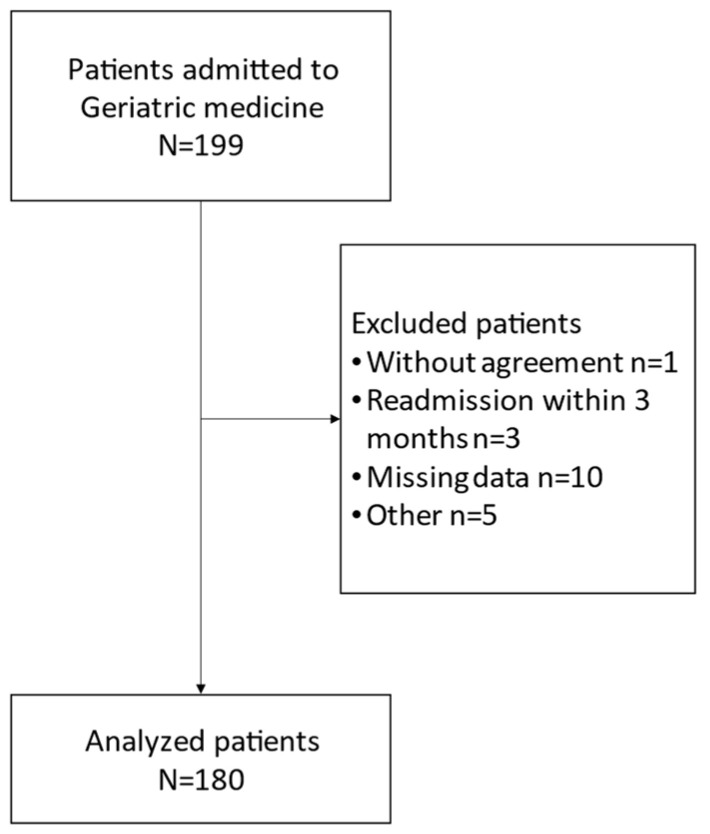
Flow chart of the final study cohort.

In this study, 199 patients were initially registered in the database for analysis (Figure 1). After applying the inclusion and exclusion criteria, 180 patients were ultimately included in the analysis. These patients, with an average age of 86.0 years (ranging from 38.9% males), were assessed for hospitalization-precipitating diseases, which included respiratory infections (14.2%), gastrointestinal/liver/pancreatic diseases (10.5%), nutritional support (10.5%), hematologic metabolic disorders (6.3%), cardiac diseases (4.7%), diabetes mellitus (4.2%), orthopedic diseases (3.7%), sepsis (3.7%), stroke (1.7%) and central nervous system diseases other than stroke (1.5%).

**Figure 2:**
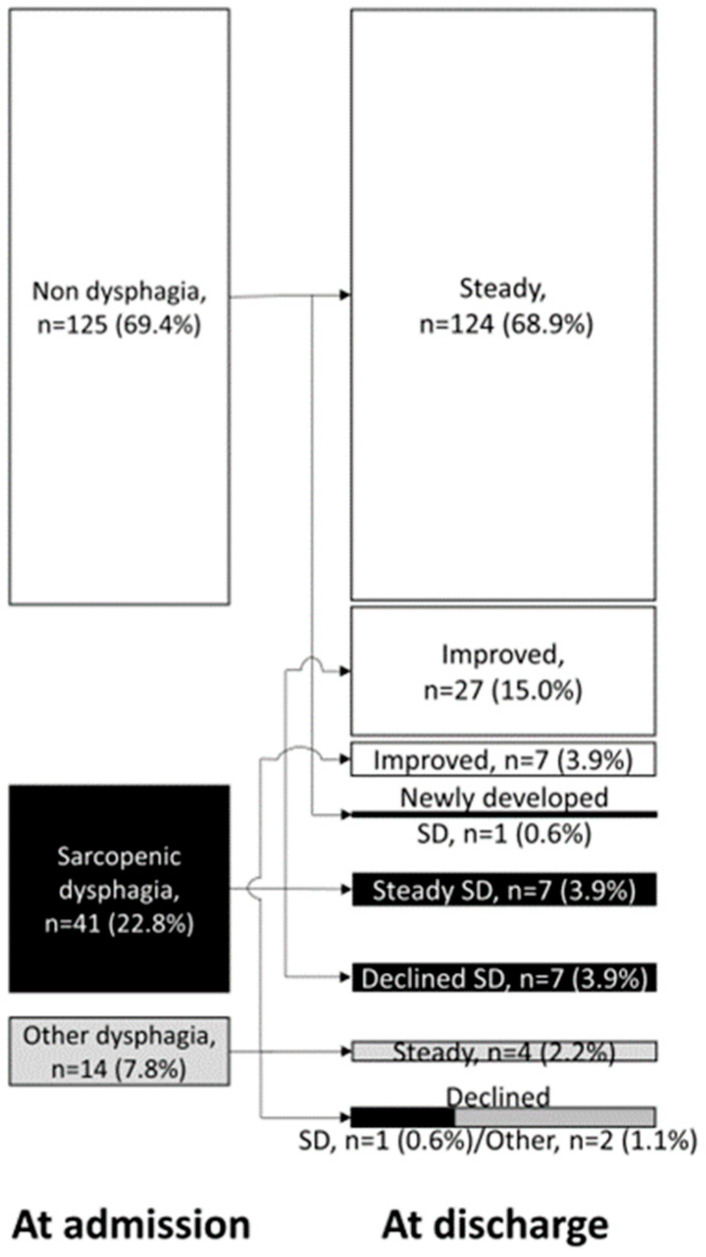
Flow chart of prevalence and course of dysphagia. Footnote: This is a flowchart depicting the progression of dysphagia during hospitalization. On the left side, the prevalence of dysphagia at the time of admission is shown. On the right side, the prevalence of dysphagia at the time of discharge is displayed, categorized based on whether the swallowing function improved, became steady, declined, or if a new dysphagia developed during the hospital stay.

The prevalence of dysphagia among these patients was examined from admission to discharge, revealing some significant findings. The prevalence of dysphagia from admission to discharge is shown in Figure 2. Initially, 55 patients (30.6%) presented with dysphagia upon admission, with 41 of them (22.8%) diagnosed with SD and 14 (7.8%) having dysphagia due to other causes. Patients with dysphagia on admission displayed notably severe sarcopenia, poorer nutritional status, and a greater need for nursing care compared to those without dysphagia (Table 2).

At admission, 41 (22.8%) patients had SD and 14 (7.8%) exhibited other types of dysphagia. Only one patient developed new dysphagia during hospitalization, and the swallowing function declined in 5.6% of patients during their stay. Conversely, 18.9% of the patients experienced an improvement in their dysphagia during hospitalization. The numbers in Figure 2 represent the number of patients, followed by the percentage (in parentheses) they comprise of the total.

Only one patient (0.6%) had new-onset dysphagia during hospitalization, which was SD. Swallowing function worsened during hospitalization in 6.1% of the patients, and they had more sarcopenia (*p* = 0.103, effect size: −0.55) and significantly poorer nutritional status (*p* < 0.001, effect size: −0.27). In addition, they had significantly lower mean energy (*p* = 0.003, effect size: −0.21) and protein intakes (*p* = 0.002, effect size: −0.24) during the first five days of hospitalization and a longer duration of fasting (*p* = 0.009, effect size: −0.22) (Table 3).

Dysphagia improved during hospitalization in 18.9% of the patients, especially those with SD who recovered more quickly than other types of dysphagia (*p* < 0.001). Patients with improved dysphagia had a significantly better nutritional status (*p* = 0.015, effect size: 0.33) and higher BI score (*p* = 0.001, effect size: 0.47) on admission than those without improved dysphagia, but there were no significant differences in other parameters. However, there was only a small effect on protein intake (effect size: 0.24) (Table 4).

## 4. Discussion

In this study, we determined the prevalence of SD and the course and characteristics of dysphagia during hospitalization in older adults admitted to the geriatric department of a specialized medical center. We found that in geriatric patients, compared to other types of dysphagia, the prevalence of SD (22.8%) is about three times higher, and SD may show signs of improvement.

It is well established that sarcopenia is highly prevalent among older individuals [28] and can often lead to dysphagia. Consequently, in the context of hospitalized elderly patients, dysphagia is frequently attributed to SD. For instance, all cases of dysphagia observed in older female patients following orthopedic surgery were linked to sarcopenia [18]. Another potential cause of SD is dysphagia during stroke recovery [29], and there is also a connection between muscle mass and dysphagia in cases of acute congestive heart failure (CHF) [30]. Therefore, sarcopenia emerges as a common underlying factor in dysphagia in this study, with SD being three times more prevalent than dysphagia due to other causes. When dysphagia is examined in the context of sarcopenia, there are important considerations to be made. Sarcopenia may potentially cause dysphagia, while dysphagia could lead to dietary restrictions, resulting in decreased muscle mass and the development of sarcopenia. Based on our data, we cannot assert a causal relationship between dysphagia and sarcopenia, and further research is required to establish these associations. In future studies, it will be essential to track the temporal relationship between sarcopenia and dysphagia, investigating in detail which condition is more likely to manifest first. Furthermore, it is crucial to delve into the mechanisms and physiological processes through which dysphagia may induce sarcopenia. This may offer new perspectives for improving clinical interventions and treatment strategies. Consequently, healthcare providers should direct their attention towards SD in older adults, given that dysphagia has a profound impact on their quality of life and necessitates appropriate care. 

Compared to other types of dysphagia, SD may show signs of improvement. Sarcopenia is a reversible condition, and similarly, SD can be ameliorated through rehabilitation therapy and robust nutritional management [14,31,32,33,34]. Our study revealed that patients who exhibited improvement in dysphagia consumed more energy and protein compared to those who did not experience improvement. While the differences between the two groups were not statistically significant, a moderate effect size (0.2) for protein intake hinted at a potential correlation, albeit constrained by our relatively small sample size. The management of sarcopenic dysphagia necessitates a comprehensive, multi-disciplinary approach. Various interventions are employed to disrupt the harmful cycle between dysphagia and malnutrition. These interventions encompass strengthening the swallowing muscles, implementing physical therapy, utilizing occupational therapy, providing nutritional support, and adjusting food texture. The geriatric medicine approach for caring for older adults involves a holistic intervention encompassing appropriate nutritional management and rehabilitation, which likely contributes to the observed recovery from SD. Interestingly, contrary to our findings, a prior report suggested that SD is less likely to improve compared to other types of dysphagia [17]. This discrepancy may arise from differences in the patient population studied, with previous reports focusing on patients with dysphagia in acute care hospitals. This may have included a relatively large number of patients (17%) who would be expected to recover to some extent over time, such as patients with acute stroke. Additionally, variations in care policies for elderly patients with dysphagia between different medical specialties may account for these differing outcomes. However, this study has a descriptive nature, and the comparative data have been collected from the literature. Our study had a limited sample size, which was insufficient to conduct a detailed analysis of swallowing disorders beyond SD. Therefore, this study does not adequately support claims of a higher prevalence or better prognosis. In future research, we believe that larger-scale comparative studies are necessary to provide more reliable results. There may be differences in prognosis and treatment approaches between SD and other types of swallowing disorders. We believe it is important to investigate the underlying causes and etiology of swallowing disorders.

Dysphagia is expected to improve in geriatric medicine. Geriatric medicine specializes in addressing the unique healthcare needs of older adults and is adept at diagnosing and managing age-related diseases. The health concerns of older individuals often entail distinctive issues and complex factors not commonly encountered in the general adult population. Hence, geriatricians possess specialized knowledge and experience in addressing the diseases and health risks peculiar to older adults, ensuring optimal treatment and care. Geriatricians collaborate closely with multidisciplinary teams comprising medical professionals, nurses, and caregivers to enhance the health and quality of life of older individuals [35]. Notably, the Asian Working Group of Sarcopenia has reported that geriatricians are more inclined to assess and intervene in sarcopenia compared to other physicians, further emphasizing the potential impact of geriatric medicine on improving swallowing function in patients [36]. In this study, there was only one new-onset case of SD during hospitalization [16,18]. This suggests that the geriatric medicine approach based on CGA may also help prevent dysphagia. The risk factors of SD include hospitalization, low physical function, malnutrition, and severe sarcopenia. In this study, patients underwent comprehensive assessments based on the CGA, followed by targeted interventions. All patients with impaired Activities of Daily Living initiated rehabilitation and received interventions for oral health issues. Furthermore, nutritional assessments and management were conducted by specialized nutrition teams. The combined approach of rehabilitation, nutrition, and oral interventions has proven to be an effective means of improving swallowing function [37]. 

Swallowing difficulty and CGA may have a significant relevance in the management of elderly individuals’ health. Swallowing difficulty is a common health issue among older adults, affecting various aspects such as physical, nutritional, and psychological well-being. Comprehensive geriatric assessment serves as a valuable tool for comprehensively evaluating the health status of older adults and creating appropriate care plans. Comprehensive geriatric assessment evaluates not only nutritional status and swallowing function but also cognitive function, mental health, and social support in the overall health assessment of older adults. This underscores the importance of nutritional management and rehabilitation in developing individualized care plans for elderly individuals. Therefore, the implementation of a comprehensive geriatric assessment may play a significant role in improving swallowing difficulties in older adults and serve as an integral part of comprehensive care.

The key strength of our study lies in shedding light on the previously underreported prevalence rates of SD. Awareness of its prevalence is crucial as it aids in identifying high-risk populations, potentially leading to preventive measures and early interventions. This insight can also serve as evidence for the necessity of providing effective care in facilities and wards with a substantial number of older inpatients, thereby advocating for appropriate staffing and training. Additionally, it contributes to enhancing the understanding of dysphagia among patients and their families, emphasizing the importance of early diagnosis and treatment.

Nonetheless, our study is not without limitations. The relatively small sample size prevented us from conducting a multivariate analysis. However, it is worth noting that nutritional status, energy intake, and protein intake may play a role in the prognosis of dysphagia, offering opportunities for intervention. In this study, we did not conduct tests such as FEES or VFS on all participants to evaluate the prevalence of dysphagia. Depending on the assessment method used, there is a possibility that dysphagia could be underestimated, or the risk assessment for aspiration and silent aspiration might be inadequate [38,39,40,41]. However, by evaluating swallowing function in a multifaceted manner with a multidisciplinary team well versed in the assessment of dysphagia, we minimized the risk of overlooking or underestimating dysphagia. Considering that FEES and VFS are recognized as the gold standard, there is also a limitation in that we only conducted these assessments on a subset of patients. However, in actual clinical settings, we believe it is essential to make the best judgments while considering resources, time, and the patient’s condition. In addition, we did not have data on complications related to dysphagia during hospitalization, such as aspiration pneumonia. It is known that dysphagia has a higher risk of leading to aspiration pneumonia, and the occurrence of aspiration pneumonia impacts prognosis and costs. Therefore, it is necessary to focus on the occurrence of complications during the treatment course of dysphagia. Moreover, our study was limited to a single geriatric ward, which means that the prevalence and prognosis of SD reported here may not necessarily generalize to general acute care wards. Nevertheless, the finding that over 20% of patients in the geriatric ward exhibited SD highlights the importance of raising awareness of SD in older hospitalized patients. Future perspectives could benefit from multicenter studies to gain a more comprehensive understanding of the association with long-term prognosis.

## 5. Conclusions

The prevalence of SD in patients admitted to the geriatric ward was 22.8%, and SD may show signs of improvement compared to other type of dysphagia. Nutritional therapy may aid in the recovery from SD, and a comprehensive care policy based on CGA may be helpful in the treatment of dysphagia. Therefore, further studies are warranted. Our findings will help identify individuals at high risk for SD early, thereby allowing preventive approaches to be taken. In addition, knowledge of the course of SD and its characteristics may lead to new effective therapeutic approaches.

## Figures and Tables

**Table 1:** Assessment and intervention by comprehensive geriatric assessment.

Assessment	
Swallowing function	Food texture, posture, oral condition, denture condition, eating habits, and preferences
Nutritional aspects	Nutritional status, normal body weight and weight change, amount of food intake and time required, excretion conditions, and bedsores or skin condition
Physical function	ADL, sleep rhythm, and sarcopenia
**Intervention**	
Rehabilitation	Directed immediately after admission in almost all patients
Nutritional management	Intervention by the Nutrition Support Team, a team of nutrition experts (Multidisciplinary team consisting of physicians, dietitians, nurses, pharmacists, therapists, laboratory technicians, etc.)
Oral problem	Immediate intervention by dentists and dental hygienists

**Table 2:** Characteristics of patients with dysphagia on admission.

	Non Dysphagia *n* = 125	Dysphagia *n* = 55	*p*-Value	Effect Size
Sex, male, *n* (%)	78 (62.4)	35 (61.8)	1.000	0.01
Age, years	85.6 ± 6.2	87.2 ± 6.1	0.115	0.12
FOIS, *n* (%)			<0.001	−0.89
1–3	0 (0.0)	5 (9.1)		
4	0 (0.0)	15 (27.3)		
5	0 (0.0)	35 (63.6)		
6	21 (16.0)	0 (0.0)		
7	110 (84.0)	0 (0.0)		
Sarcopenia, *n* (%)	72 (57.6)	48 (87.3)	<0.001	−0.65
CCI score	3.00 [1.00, 4.00]	3.00 [2.00, 4.00]	0.081	−0.13
MNA-SF score	9.00 [7.00, 12.00]	5.00 [4.00, 7.00]	<0.001	−0.51
Dependency level, *n* (%)			NA	NA
Independent	22 (17.6)	0 (0.0)		
Assistance	19 (15.2)	0 (0.0)		
Moderately dependent	62 (49.6)	25 (45.5)		
Highly dependent	12 (8.8)	29 (52.7)		
Not certified	10 (8.0)	1 (1.8)		

Abbreviations: FOIS, Functional Oral Intake Scale; CCI, Charlson Comorbidity Index; MNA-SF, Mini Nutritional Assessment-Short Form.

**Table 3:** Characteristics of patients with declining swallowing function during hospitalization.

	Not Declined *n* = 169	Declined *n* = 11	*p*-Value	Effect Size
Sex, male, *n* (%)	105 (62.1)	7 (63.6)	1.000	−0.03
Age, years	86.04 ± 6.22	87.09 ± 7.30	0.592	−0.03
Sarcopenia, *n* (%)	110 (65.1)	10 (90.9)	0.103	−0.55
MNA-SF, score	8.00 [6.00, 11.00]	5.00 [3.00, 5.50]	<0.001	−0.27
Mean energy intake, kcal	1031.50 ± 405.83	657.37 ± 306.37	0.003	−0.21
Mean protein intake, g	42.85 ± 18.56	24.70 ± 13.91	0.002	−0.24
Nil per os, days	0.34 ± 1.07	1.27 ± 1.85	0.009	−0.22
BI, score	55.0 [20.0, 90.0]	10 [0.0, 25.0]	<0.001	0.86
CCI, score	3.00 [1.00, 4.00]	3.00 [2.50, 3.00]	0.429	−0.06
Length of stay, days	30.51 ± 51.28	31.45 ± 26.13	0.952	0.00

Abbreviation: MNA-SF, Mini Nutritional Assessment-Short Form; BI, Barthel Index; CCI, Charlson Comorbidity Index; Nil per os, Nothing by mouth.

**Table 4:** Characteristics of patients with improvement in swallowing function during hospitalization.

	Not Improved *n* = 21	Improved *n* = 34	*p*-Value	Effect Size
Sex, male, *n* (%)	11 (52.4)	23 (67.6)	0.392	−0.31
Age, years	86.14 ± 7.19	87.88 ± 5.45	0.314	−0.16
Sarcopenia (%)	19 (90.5)	29 (85.3)	0.696	0.15
MNA-SF, score	5.00 [3.00, 6.00]	6.00 [4.25, 8.00]	0.015	0.33
Mean energy intake, kcal	796.52 ± 329.74	843.64 ± 389.00	0.646	0.01
Mean protein intake, g	31.29 ± 14.08	33.12 ± 14.62	0.649	0.24
Nil per os, days	0.76 ± 1.45	0.53 ± 1.19	0.519	0.06
BI, score	0.00 [0.00, 15.00]	15.00 [5.00, 42.50]	0.001	0.47
CCI, score	3.00 [2.00, 4.00]	3.00 [2.00, 4.75]	0.916	0.13
Length of stay, days	63.29 ± 106.61	28.38 ± 18.37	0.066	0.25

Abbreviation: MNA-SF, Mini Nutritional Assessment-Short Form; BI, Barthel Index; CCI, Charlson Comorbidity Index; Nil per os, Nothing by mouth.

## Data Availability

Data is unavailable due to privacy and ethical restrictions.
